# Pulmonary Embolism After Acute Ischaemic Stroke (PEARL-AIS): Global Prevalence, Risk Factors, Outcomes, and Evidence Grading from a Meta-Analysis

**DOI:** 10.3390/neurolint17100168

**Published:** 2025-10-12

**Authors:** Darryl Chen, Yuxiang Yang, Sonu M. M. Bhaskar

**Affiliations:** 1 Global Health Neurology Lab, Sydney, NSW 2150, Australia; 2 UNSW Medicine and Health, South West Sydney Clinical Campuses, University of New South Wales (UNSW), Sydney, NSW 2170, Australia; 3 NSW Brain Clot Bank, NSW Health Pathology, Sydney, NSW 2170, Australia; 4 Clinical Sciences Stream, Ingham Institute for Applied Medical Research, Liverpool, NSW 2170, Australia; 5 Department of Neurology & Neurophysiology, Liverpool Hospital and South West Sydney Local Health District, Liverpool, NSW 2150, Australia; 6 Division of Cerebrovascular Medicine and Neurology, Department of Neurology, National Cerebral and Cardiovascular Center (NCVC), Suita 564-8565, Osaka, Japan

**Keywords:** pulmonary embolism, acute ischaemic stroke, venous thromboembolism, thromboprophylaxis, meta-analysis, thromboinflammatory axis, evidence grading

## Abstract

*Objectives:* Pulmonary embolism (PE) is an uncommon but potentially fatal complication of acute ischaemic stroke (AIS). Its global burden and prevention remain incompletely defined. We performed a systematic review and meta-analysis (PEARL-AIS) to estimate prevalence, risk factors, outcomes, and prophylactic efficacy, with GRADE evidence appraisal. *Methods*: Following PRISMA 2020 and MOOSE guidelines, five databases (PubMed, Embase, Cochrane, Scopus, Web of Science) were searched (1995–2024). The protocol was prospectively registered (OSF s25ny). Random-effects models (DerSimonian–Laird; REML sensitivity) were used to pool prevalence and odds ratios; heterogeneity was evaluated with I^2^, Cochran’s Q, and τ^2^. Influence (leave-one-out) and subgroup analyses for prevalence and mortality of PE in AIS were explored. Bias was assessed using the Modified Jadad Scale; overall certainty was graded with the GRADE framework. *Results*: Twenty-four studies met the inclusion criteria (*n* = 25,666,067), of which seventeen studies (*n* = 23,637,708) contributed to pooled prevalence analyses. The pooled prevalence of PE after AIS was 0.40% (95% CI 0.33–0.49), approximately six-fold higher than in the general population, with considerable heterogeneity (I^2^ > 90%, Cochrane classification). The pooled mortality among AIS patients with PE was 12.9% (95% CI 1.6–31.7). Mortality risk was significantly higher in AIS patients with PE (OR 4.96, 95% CI 2.98–8.24). Atrial fibrillation (29%), cancer (19%), and smoking (23%) were common; hypertension (54%) and diabetes (23%) were prevalent but not predictive, with diabetes showing a paradoxical protective association (OR 0.88, 95% CI 0.84–0.92). Pharmacological prophylaxis was associated with a reduced risk of PE (OR 0.64, 95% CI 0.46–0.90; I^2^ = 0%), supported by moderate-certainty evidence. *Conclusions*: PE is an uncommon but often fatal complication of AIS. Traditional venous thromboembolism predictors underperform in this context, suggesting a stroke-specific thromboinflammatory mechanism linking the brain and lung axis. Despite considerable heterogeneity and low-to-moderate certainty of evidence, pharmacological prophylaxis demonstrates a consistent protective effect. Systematic PE surveillance and tailored prophylactic strategies should be integral to contemporary stroke care, while future studies should refine risk stratification and elucidate the mechanistic underpinnings of this *brain–lung thromboinflammatory* continuum.

## 1. Background

Pulmonary embolism (PE) represents one of the most devastating complications of acute ischaemic stroke (AIS), amplifying an already high burden of morbidity and mortality [1]. As a manifestation of venous thromboembolism (VTE), PE contributes to in-hospital deaths and often escapes timely recognition due to overlapping neurological and systemic symptoms [2]. In the general population, the annual prevalence of PE is estimated at 0.05–0.07% [3,4,5]; however, evidence suggests that stroke patients carry a markedly elevated risk [6], attributed to prolonged immobility [7,8], endothelial dysfunction [9,10,11], systemic inflammation [12], and prothrombotic cascades. Despite these clinical concerns, the true prevalence of PE after AIS and its predictors remains insufficiently defined.

Over recent decades, advances in reperfusion therapies, intravenous thrombolysis (IVT) and endovascular thrombectomy (EVT) have transformed acute stroke outcomes [13]. Yet, their influence on VTE risk remains unclear, particularly as treatment-related factors (immobilisation, critical illness, haemorrhagic risk) complicate thromboprophylaxis decisions [14,15]. Although pharmacological and mechanical prophylaxis are routinely recommended [16,17,18], their real-world efficacy in stroke populations has not been systematically quantified. Importantly, concerns about haemorrhagic transformation often temper anticoagulant use [19], leaving clinicians uncertain about the optimal balance between preventing PE and minimising bleeding complications.

Beyond epidemiology, emerging data point to novel mechanistic pathways linking brain injury to peripheral thrombosis [20]. Stroke is increasingly recognised as a systemic disease with immune and neurohumoral sequelae that extend beyond the brain [21,22,23,24]. Neutrophil extracellular traps (NETs) [25,26], cytokine surges (e.g., IL-6, TNF-α) [9,10,11], and brain-derived extracellular vesicles carrying procoagulant microparticles may converge on the pulmonary vasculature, creating a fertile ground for in situ thrombosis even in the absence of deep vein thrombosis (DVT) [27,28]. This proposed *brain–lung thromboinflammatory axis* [6] reframes PE in AIS as not merely an embolic complication but as part of a broader systemic cascade, analogous to immunothrombosis seen in sepsis and COVID-19.

Despite its clinical importance, knowledge gaps persist in three areas: (1) the true global prevalence and regional variability of PE in AIS patients, (2) the prognostic impact of PE on mortality and functional outcomes, and (3) the effectiveness and safety of prophylactic strategies tailored to this high-risk group [29]. Addressing these questions is critical to informing evidence-based guidelines and improving patient outcomes.

The *Pulmonary Embolism After Acute Ischaemic Stroke (PEARL-AIS)* study was designed to meet this need. By conducting a comprehensive meta-analysis [6], PEARL-AIS aims to synthesise global data on PE prevalence, risk factors, and outcomes in AIS, while also evaluating the protective role of prophylaxis. Moreover, by integrating clinical epidemiology with emerging mechanistic insights, this work advances the concept of a *brain–lung thromboinflammatory axis*. In doing so, it provides a comprehensive framework for future research and lays the foundation for risk-stratified prevention strategies in acute stroke care.

## 2. Methodology

### 2.1. Literature Search and Study Selection

**Figure 1:**
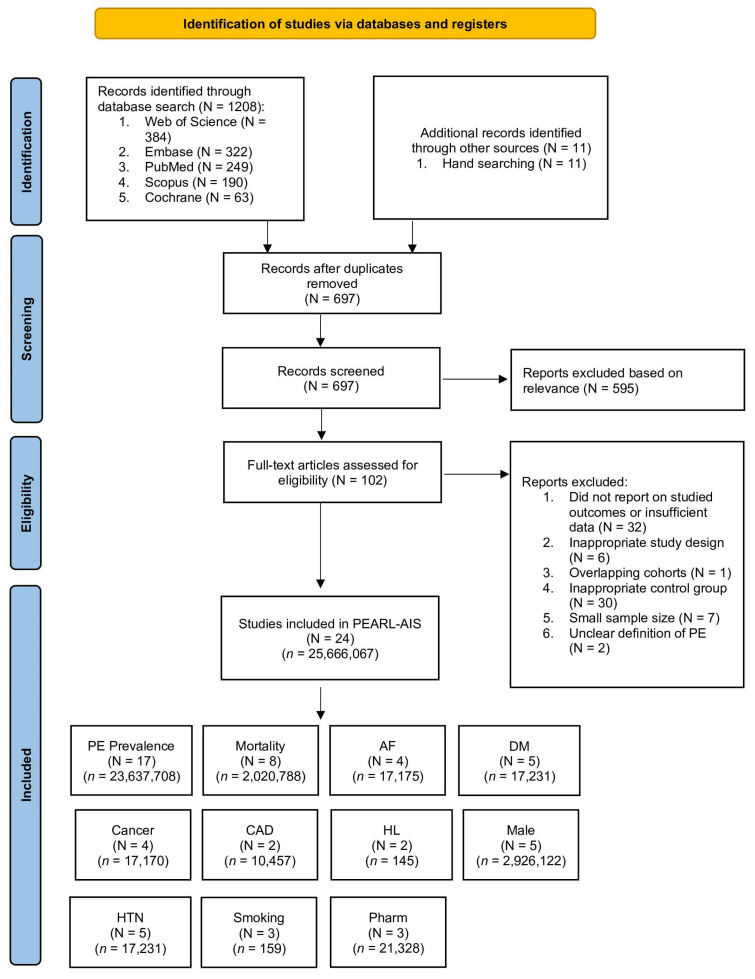
PRISMA flow diagram of study selection for the PEARL-AIS systematic review and meta-analysis. Abbreviations: AF: atrial fibrillation; CAD: coronary artery disease; DM: diabetes mellitus; HL: hyperlipidaemia; HTN: hypertension; PE: pulmonary embolism; *n*: number of patients; N = number of studies; Pharm: pharmacological prophylaxis.

We conducted this systematic review and meta-analysis in accordance with PRISMA 2020 and MOOSE guidelines. A comprehensive literature search was performed across PubMed, Embase, Cochrane Library, Scopus, and Web of Science for the period January 1995 to December 2024. Search terms combined Medical Subject Headings (MeSH) and free-text keywords, including *pulmonary embolism*, *acute ischaemic stroke*, *prophylaxis*, *risk factors*, and *endovascular thrombectomy*. Boolean operators (AND/OR) were applied, and detailed search strategies are provided in the Supplemental Information (SI). We additionally hand-searched reference lists of systematic reviews, meta-analyses, and key primary studies to identify eligible articles not captured in the database search. The review protocol was prospectively registered with the Open Science Framework (OSF) (Registration No. s25ny, https://osf.io/s25ny/). The PRISMA flow diagram of study selection is shown in Figure 1. Compliance with PRISMA and MOOSE reporting guidelines is documented in Supplemental Information Tables S1 and S2.

### 2.2. Inclusion and Exclusion Criteria

Studies were included if they: (1) enrolled adult patients (≥18 years) with acute ischaemic stroke; (2) reported cases of pulmonary embolism; (3) provided data on prevalence, risk factors, outcomes, or prophylaxis; (4) included ≥ 20 patients; and (5) were published in English between 1995 and 2024. Exclusion criteria were animal studies, case reports/series with insufficient data, reviews, editorials, and studies lacking full-text availability. Duplicate records were removed.

### 2.3. Data Extraction

Data extraction was performed independently by two reviewers using a standardised form, with discrepancies resolved by consensus or by consulting a third reviewer. Extracted information included study characteristics (author, year, country, design, sample size, inclusion criteria), patient demographics (age, sex, comorbidities), and clinical characteristics such as prophylaxis strategies. A summary of the clinical and demographic characteristics of included studies is provided in Table 1. Outcomes of interest included prevalence of pulmonary embolism, mortality, length of stay, and the pooled prevalence of comorbidities such as atrial fibrillation, hypertension, diabetes mellitus, smoking, cancer, coronary artery disease, and hyperlipidaemia. Predictors of pulmonary embolism and outcomes were extracted as odds ratios (ORs) or relative risks (RRs) with corresponding 95% confidence intervals (CIs). For each study, we also extracted variables required for moderator analyses: study design, geographic region (continent), and PE ascertainment approach (routine imaging vs. clinical coding). PE ascertainment methods and follow-up windows for each study are detailed in Supplemental Information Table S6.

### 2.4. Ethics Statement

This study was a systematic review and meta-analysis of previously published data and did not involve direct interaction with human subjects. As such, it did not constitute human subjects research and institutional review board (IRB) approval and informed consent were not required.

### 2.5. Methodological Quality Assessment of Included Studies

Methodological quality of the included studies was rigorously assessed. Study quality was appraised using a modified Jadad scale [30] adapted for non-interventional observational studies. ROBINS-I was not applied due to the absence of intervention-related parameters across the included datasets. Studies judged to be at high risk of bias were excluded from quantitative synthesis but were discussed narratively if they offered relevant insights. Funding-related bias assessments are provided in Supplemental Information Table S4.

### 2.6. Statistical Analysis

All statistical analyses were conducted using STATA/MP version 13.0 (StataCorp LLC, College Station, TX, USA). Pooled prevalence estimates for PE and comorbidities were calculated using the metaprop command, reported with 95% CIs. Associations between predictors and outcomes were analysed as ORs or RRs with 95% CIs using the metan command. Subgroup analyses were prespecified by study design (Prospective Vs. Retrospective) and geographic region (Asia, Europe, North America, Africa, multi-country). Heterogeneity across studies was assessed using Cochran’s Q, I^2^, H, and τ^2^ statistics. I^2^ values of 0–40%, 30–60%, 50–90%, and 75–100% were interpreted as low, moderate, substantial, and considerable heterogeneity, respectively, following the Cochrane Handbook [31]. Tests of overall effect were derived from Z-statistics and corresponding *p*-values. Sensitivity analyses, including leave-one-out procedures, were performed to examine the robustness of pooled estimates. Using the metainf command, each study was sequentially excluded to assess its influence on the overall effect. Publication bias was evaluated using Egger’s test of effect sizes. Pharmacological prophylaxis analyses were conducted using pooled ORs comparing treated versus untreated groups and assessed for heterogeneity using random-effects models. Forest plots were generated to visualise pooled outcomes. A *p*-value < 0.05 was considered statistically significant.

**Table 1:** Clinical characteristics of studies included in the PEARL-AIS meta-analysis.

Author (Year)	Country	Study Design	Mean Age (Years, SD)	PE (*n*)	PE (%)	Male (%)	Mortality (*n*, %)
Abdelsalam et al. (2020) [32]	Egypt	Prospective	–	9	5.69	–	–
Ahmed et al. (2023) [33]	USA	Retrospective	–	26,758	0.50	–	–
Ali et al. (2009) [34]	Multiple	Retrospective	74.7 (9.7)	22	4.14	53	–
Allendorfer et al. (2007) [35]	Germany	Prospective	54.3 (19.2)	–	–	–	–
Amin et al. (2013) [36]	USA	Retrospective	62.2 (12.2)	5	0.33	–	–
CAST Collaboration Group (1997) [37]	China	Prospective	–	32	0.15	–	15 (PE), 726 (no PE)
Che et al. (2024) [38]	China	Prospective	–	4	1.31	–	–
Chen et al. (2012) [39]	Taiwan	Retrospective	70.1 (16.8)	–	–	–	5 (PE), 9 (no PE)
Dennis et al. (2011) [40]	Multiple	Prospective	75.3 (11.9)	75	1.33	49	–
Eswaradass et al. (2018) [41]	Canada	Retrospective	–	10	0.32	–	–
Huang et al. (2021) [42]	China	Retrospective	–	1743	0.21	–	55 (PE), 1688 (no PE)
IST Collaborative Group (1997) [43]	–	Prospective	–	–	–	–	–
Keller et al. (2024) [44]	Germany	Retrospective	–	–	–	46	1938 (PE), 4766 (no PE)
Keller et al. (2020) [45]	Germany	Retrospective	75.3 (11.9)	10,368	0.36	–	2943 (PE), 7425 (no PE)
Kelly et al. (2004) [46]	USA	Prospective	70.1 (11.9)	12	11.54	46	–
Pongmoragot et al. (2013) [47]	Canada	Retrospective	–	89	0.79	52	28 (PE), 61 (no PE)
Sherman et al. (2007) [48]	Multiple	Prospective	–	7	0.52	–	–
Skaf et al. (2005) [49]	USA	Retrospective	–	72,000	0.51	–	–
Skaf et al. (2006) [50]	USA	Retrospective	–	–	–	–	11,101 (PE), 1,989,862 (no PE)
Sluis et al. (2021) [51]	Multiple	Retrospective/Prospective	68.7 (13.4)	8	21.05	64	–
Sprigg et al. (2005) [52]	UK	Prospective	73.3 (10.4)	20	1.35	54	–
Tanislav et al. (2011) [53]	Germany	Prospective	55.2	–	–	57	–
TOAST Investigators (1998) [54]	USA	Prospective	65.5 (11.4)	6	0.47	–	–
Turpie et al. (2013) [55]	Multiple	Prospective	–	–	–	56	10 (PE), 156 (no PE)

Note: Dashes (–) indicate missing or unreported demographic data in the original studies. The mixed-design study reporting “Prospective + retrospective” represents a study with both prospective and retrospective components, and pooled prevalence analysis was generated without this outlier (21.05%). Abbreviations: *n* = number of patients; PE = pulmonary embolism; SD = standard deviation.

### 2.7. Evidence Grading

The certainty of evidence across all primary and secondary outcomes was appraised using the GRADE (*Grading of Recommendations*, *Assessment*, *Development*, *and Evaluation*) framework [56]. Five domains, risk of bias, inconsistency, indirectness, imprecision, and publication bias, were systematically assessed. Evidence was categorised as high, moderate, low, or very low certainty. RCTs were initially considered high-certainty evidence, while observational studies were considered low-certainty, with ratings adjusted according to study quality, effect magnitude, and heterogeneity. To ensure clarity and avoid duplication, only predictors with clinically meaningful and/or statistically significant associations were carried forward into the GRADE evidence profiles. Where both prevalence and predictor analyses were available for a comorbidity, only predictor analyses (odds ratios) were included. A GRADE Summary of Findings table was then constructed to provide an overview of the certainty of evidence for pooled prevalence, key risk factors, mortality outcomes, and the effectiveness of prophylactic strategies.

## 3. Results

A total of twenty-four studies met the inclusion criteria, encompassing 25,666,067 patients overall; among these, seventeen studies (*n* = 23,637,708) contributed to the pooled prevalence analyses of PE following AIS.

### 3.1. Prevalence of Pulmonary Embolism

**Figure 2:**
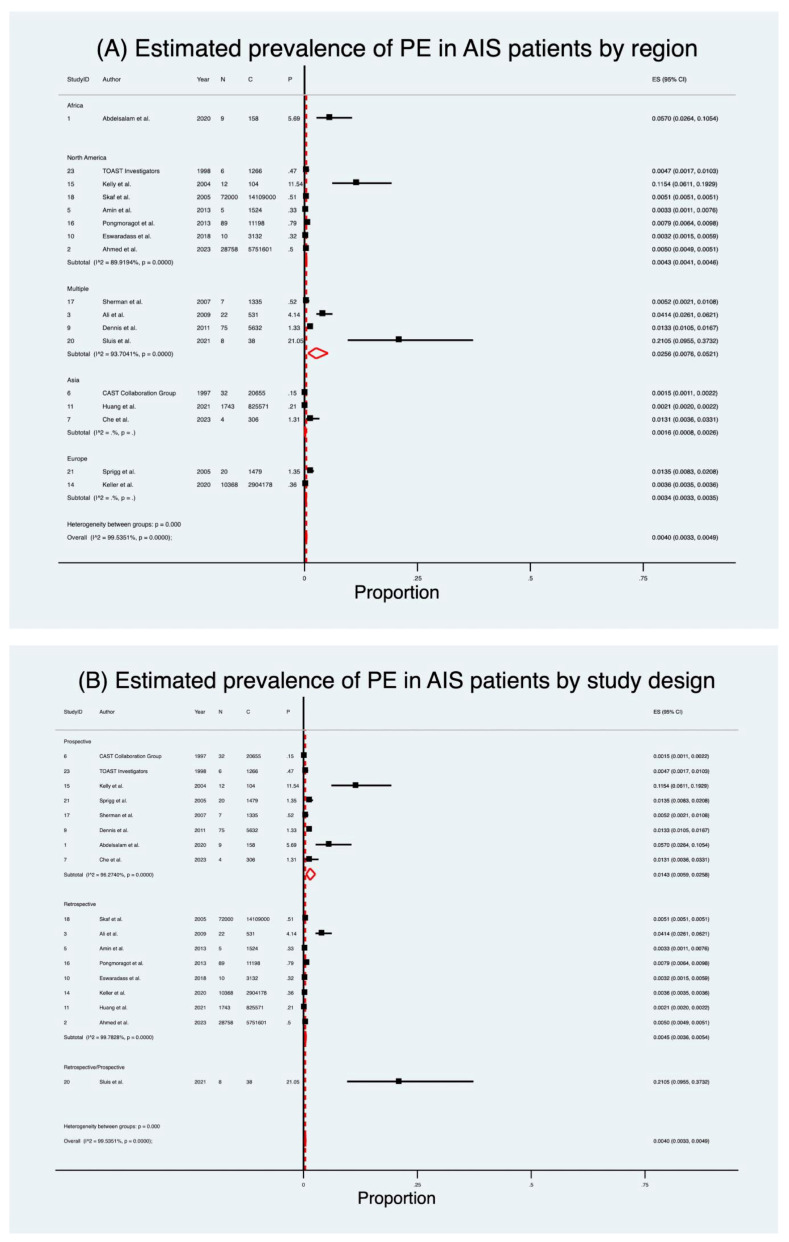
Forest plot of pooled prevalence of pulmonary embolism following acute ischaemic stroke [32,33,34,36,37,38,40,41,42,45,46,47,48,49,51,52,54], stratified by: (**A**) study design and (**B**) geographic region. Black squares represent individual study estimates with 95% CIs (horizontal lines), and red diamonds indicate the pooled estimates with corresponding CIs. Abbreviations: AIS: acute ischaemic stroke; C: total number of patients; CI: confidence interval; ES: effect size; P: proportion of patients with outcome; PE: pulmonary embolism; N: number of patients with outcome.

Seventeen studies [32,33,34,36,37,38,40,41,42,45,46,47,48,49,51,52,54] encompassing 23,637,708 patients were included in the pooled analysis of PE prevalence after AIS (Table 2; Figure 2). Details of PE and follow-up duration across included studies are provided in Supplemental Information Table S6. The estimated pooled prevalence was 0.40% (95% CI 0.33–0.49), with a crude prevalence of 0.48%. Considerable heterogeneity was present (I^2^ = 99.5%, *p* < 0.001). Excluding the high-prevalence Sluis et al. [51] cohort increased the pooled prevalence to 0.46% (95% CI 0.38–0.54) with no substantial effect on the overall pooled estimates.

When stratified by study design, retrospective studies [33,34,36,41,42,45,47,49] (*n* = 23,606,735) yielded a prevalence of 0.45% (95% CI 0.36–0.54), while prospective studies [32,37,38,40,46,48,52,54] (*n* = 30,935) demonstrated a substantially higher prevalence of 1.43% (95% CI 0.59–2.58). A single mixed-design study [51] reported a crude prevalence of 21%. Subgroup heterogeneity was significant (Q = 34.14, *p* < 0.001).

Geographic variation was striking (Table 2; Figure 2). Prevalence was lowest in Asia [37,38,42] (0.16%; 95% CI 0.08–0.26) and Europe [45,52] (0.34%; 95% CI 0.33–0.35), intermediate in North America [33,36,41,46,47,49,54] (0.43%; 95% CI 0.41–0.46), and highest in multi-country cohorts [34,40,48,51] (2.56%; 95% CI 0.76–5.21). One African study [32] reported a crude prevalence of 5.7%. Subgroup heterogeneity was again significant (Q = 55.13, *p* < 0.001).

### 3.2. Mortality in AIS Patients with Pulmonary Embolism

**Figure 3:**
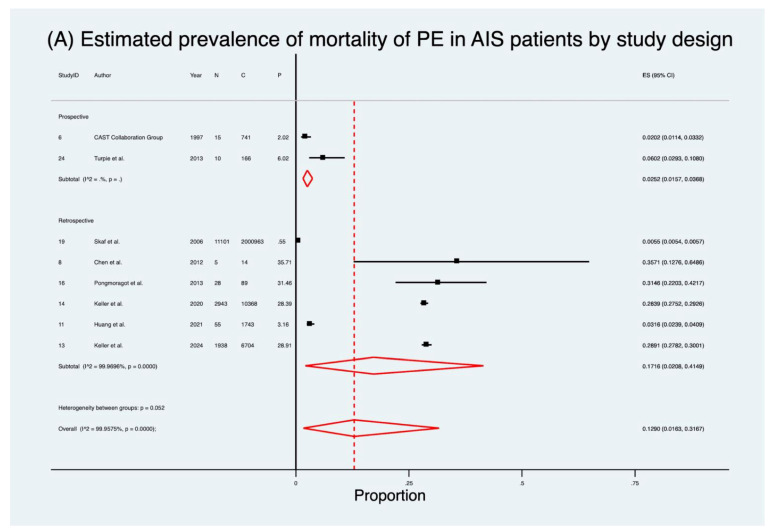

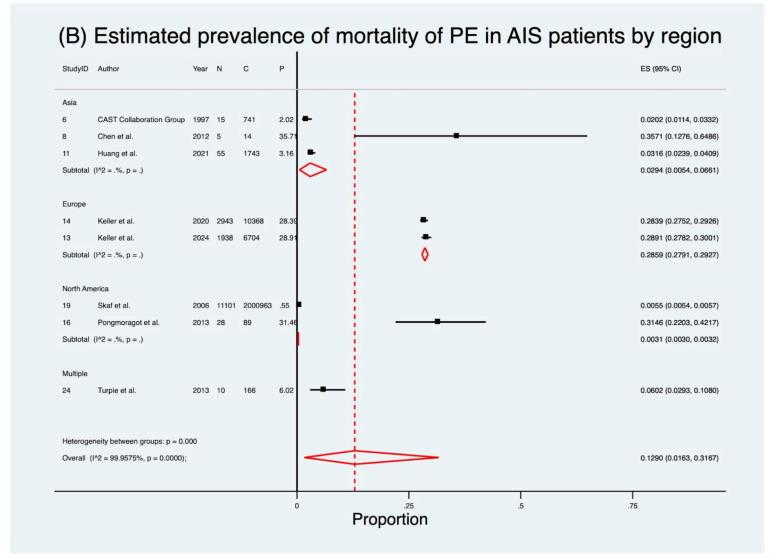
Forest plot of mortality in patients with pulmonary embolism after acute ischaemic stroke [37,39,42,44,45,47,50,55], stratified by: (**A**) study design and (**B**) geographic region. Black squares represent individual study estimates with 95% CIs (horizontal lines), and red diamonds indicate the pooled estimates with corresponding CIs. Abbreviations: AIS: acute ischaemic stroke; PE: pulmonary embolism; C: total number of patients; CI: confidence interval; ES: effect size; P: proportion of patients with outcome; N: number of patients with outcome.

Eight studies [37,39,42,44,45,47,50,55] (2,020,788 patients) evaluated mortality in AIS patients who developed PE (Table 2; Figure 3). The pooled prevalence of mortality was 12.9% (95% CI 1.6–31.7), markedly higher than the crude rate of 0.8%. Heterogeneity was considerable (I^2^ = 100.0%, *p* < 0.001). Mortality rates varied significantly by study design. Retrospective studies [39,42,44,45,47,50] demonstrated a pooled mortality prevalence of 17.2% (95% CI 2.1–41.5), compared to 2.5% (95% CI 1.6–3.7) in prospective cohorts [37,55]. Regional analysis showed the highest mortality in Europe [44,45] (28.6%; 95% CI 27.9–29.3), followed by Asia [37,39,42] (2.9%; 95% CI 0.5–6.6), with North America [47,50] reporting only 0.31% (95% CI 0.30–0.32).

### 3.3. Predictive Indicators of Pulmonary Embolism

**Figure 4:**
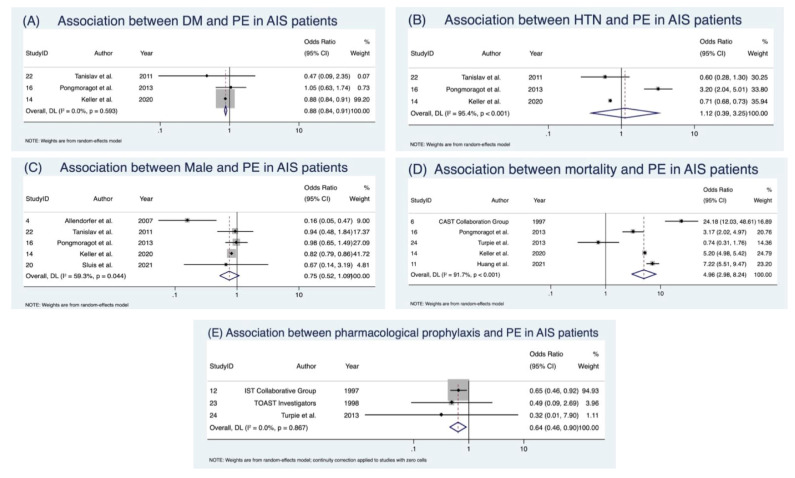
Forest plots of predictive indicators and clinical outcomes associated with pulmonary embolism after acute ischaemic stroke [35,45,47,51,53]. (**A**) DM, (**B**) HTN, (**C**) Male, (**D**) Mortality, (**E**) Pharmacological Prophylaxis. Black squares represent individual study estimates with 95% CIs (horizontal lines), and purple diamonds indicate the pooled estimates with corresponding CIs. Abbreviations: AIS: acute ischaemic stroke; CI: confidence interval; DL: DerSimo ian and Laird method; DM: diabetes mellitus; HTN: hypertension; PE: pulmonary embolism.

The discrete predictive markers assessed across studies are outlined in Table 3. Meta-analysis of discrete predictors revealed that male sex [35,45,47,51,53] was not significantly associated with PE (OR 0.75; 95% CI 0.52–1.09), though heterogeneity was moderate (I^2^ = 59.3%), consistent with Cochrane thresholds, and publication bias was suggested (Supplemental Information Figure S2; detailed outputs in Supplemental Information Table S5). Hypertension [45,47,53] was associated with a non-significant increase in odds (OR 1.12; 95% CI 0.39–3.25), with very considerable heterogeneity (I^2^ = 95.4%). Conversely, diabetes mellitus [45,47,53] showed a paradoxical protective effect, with significantly lower odds of PE (OR 0.88; 95% CI 0.84–0.92) and no/low heterogeneity (I^2^ = 0%) (Table 4; Figure 4). Detailed sensitivity analyses are presented in Supplemental Information Figure S3.

### 3.4. Clinical Outcomes Following Pulmonary Embolism

Five studies [37,42,45,47,55] (3,774,118 patients) analysed the impact of PE on AIS outcomes (Table 4; Figure 4). PE was associated with a nearly five-fold increased risk of mortality (OR 4.96; 95% CI 2.98–8.24, *p* < 0.001), with considerable heterogeneity (I^2^ = 91.7%).

### 3.5. Prevalence of Risk Factors in AIS Patients with PE

Pooling across available studies, atrial fibrillation [39,44,45,47] was present in 29% (95% CI 25–35) of AIS patients with PE (Table 2; Supplemental Information Figure S1). Other prevalent comorbidities included hypertension [39,44,45,47,53] (54%; 95% CI 49–60), diabetes mellitus [39,44,45,47,53] (23%; 95% CI 20–26), cancer [32,44,45,47] (19%; 95% CI 13–25), smoking [39,47,53] (23%; 95% CI 12–37), coronary [45,47] artery disease (15%; 95% CI 15–16), and hyperlipidaemia [47,53] (20%; 95% CI 14–27). Notably, atrial fibrillation and cancer were more common than in unselected AIS cohorts, whereas hypertension and diabetes, well-established vascular risk factors, did not appear predictive in pooled analyses (Table 4). Sensitivity analyses, including leave-one-out procedures, are detailed in Supplemental Information Figure S3. Egger’s regression outputs are provided in Supplemental Information Figure S2.

### 3.6. Pharmacological Prophylaxis

Three studies [43,54,55] (21,090 patients) assessed prophylactic interventions (Table 4; Supplemental Information Figure S1). Pharmacological prophylaxis significantly reduced PE risk (OR 0.64; 95% CI 0.46–0.90), with no heterogeneity (I^2^ = 0%). This finding was consistent across anticoagulants, though the agents studied varied (heparin, danaparoid, enoxaparin). Details of agent, dosing, and diagnostic confirmation are provided in Supplemental Information Table S7.

### 3.7. Evidence Grading Assessment Findings

The certainty of evidence, assessed with the GRADE framework (Table 5; Supplemental Information Table S6), ranged from moderate to very low. Prevalence estimates are presented in Table 2, while only predictor analyses (odds ratios) are carried forward into the GRADE evidence profiles (Table 5; Supplemental Information Table S6). Moderate-certainty evidence supported the association between diabetes mellitus and lower PE risk, mortality odds with PE, and the benefit of pharmacological prophylaxis. In contrast, estimates for sex, hypertension, and comorbidity prevalence were graded as low or very low due to heterogeneity, imprecision, and small study numbers. For GRADE profiling, only outcomes and predictors judged to be clinically meaningful and/or statistically significant were carried forward into the evidence tables. Predictors with non-significant associations (e.g., male sex) were excluded from GRADE tabulation but remain reported in the primary results tables for completeness. The detailed outputs (scores, bias assessments) of methodological quality of individual studies are presented in the Supplemental Information Table S3.

## 4. Discussion

The PEARL-AIS study represents the largest and most comprehensive synthesis to date on PE following AIS, encompassing over 25 million patients across multiple regions and study designs. Although relatively uncommon in absolute terms, PE occurred six times more frequently after AIS than in the general population [3,4,5] and carried a disproportionate mortality burden, with case fatality approaching 13% and odds of death nearly five times higher in affected patients [57,58]. Given the considerable heterogeneity (I^2^ > 90% for several outcomes, per Cochrane thresholds), these estimates should be viewed as exploratory signals rather than definitive population rates. Classical vascular risk factors such as sex, hypertension, and diabetes showed limited predictive value, while comorbidities, including atrial fibrillation, cancer, and smoking, were consistently more prevalent among patients who developed PE. Importantly, pharmacological prophylaxis significantly reduced risk, with consistent benefit across diverse settings. Our findings support implementing structured PE surveillance protocols, particularly in immobile stroke patients, and integrating prophylaxis systematically where bleeding risk permits [29]. These findings carry direct clinical implications: clinicians should maintain vigilance for PE in AIS patients with unexplained hypoxia, apply prophylaxis consistently in immobilised patients, and recognise atrial fibrillation, cancer, and smoking history as markers of higher risk. More broadly, the data reinforce a model in which stroke-specific immune and thromboinflammatory cascades [6], rather than classical venous thromboembolism predictors alone, drive pulmonary thrombosis risk. This highlights PE as an uncommon but clinically devastating complication of AIS and underscores the need for systematic surveillance, tailored prevention, and mechanistic research into the brain–lung axis [6]. However, conclusions must be interpreted with caution because most prevalence and comorbidity estimates were graded low or very low certainty.

**Figure 5:**
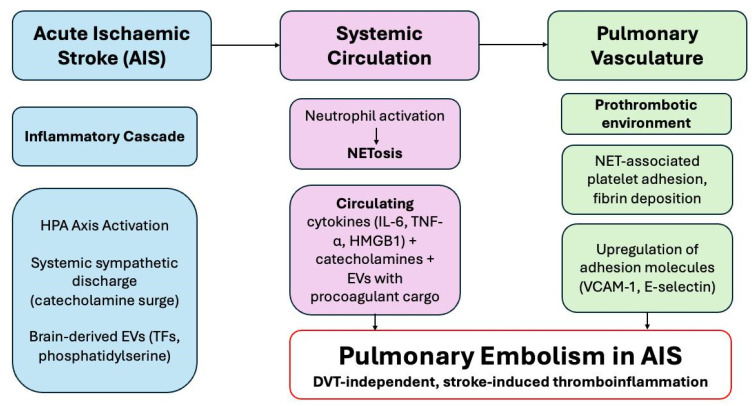
Schematic representation of the *brain–lung thromboinflammatory axis* following acute ischaemic stroke (AIS) [6]. The figure illustrates how AIS triggers systemic inflammatory and neurohumoral cascades that culminate in pulmonary embolism (PE). Following AIS, activation of the hypothalamic–pituitary–adrenal (HPA) axis and sympathetic discharge lead to systemic catecholamine surges and release of brain-derived extracellular vesicles (EVs) carrying procoagulant factors such as tissue factor (TF) and phosphatidylserine. These stimuli drive neutrophil activation and NETosis, promoting the circulation of cytokines (IL-6, TNF-α, HMGB1) and EVs that create a prothrombotic environment in the pulmonary vasculature. The resultant endothelial activation, platelet adhesion, and fibrin deposition contribute to DVT-independent, stroke-induced thromboinflammation, manifesting as pulmonary embolism after AIS. Abbreviations: AIS—acute ischaemic stroke; EVs—extracellular vesicles; HPA—hypothalamic–pituitary–adrenal; IL—interleukin; TNF-α—tumour necrosis factor-alpha; HMGB1—high mobility group box protein 1; TF—tissue factor; NETosis—neutrophil extracellular trap formation; VCAM-1—vascular cell adhesion molecule 1.

The pooled prevalence of PE after AIS was 0.40%, well above that observed in the general population (~0.05–0.07% per year) and consistent with a six-fold increased risk in stroke survivors [3,4,5]. The marked heterogeneity across studies likely reflects differences in surveillance intensity, diagnostic thresholds, and reliance on administrative versus adjudicated datasets. Prospective cohorts consistently reported higher prevalence than retrospective analyses, suggesting that underdiagnosis is common when PE is not actively sought. Mechanistically, this excess risk is compatible with the proposed *brain–lung thromboinflammatory axis* [6], in which stroke-induced immune activation—via NETosis, cytokine release, endothelial injury, and procoagulant extracellular vesicles—promotes in situ pulmonary thrombosis, even in the absence of deep vein thrombosis [23,25,59,60,61]. These findings corroborate earlier hospital-based observations of heightened VTE risk after stroke, but extend them by demonstrating that pulmonary embolism may arise independently of DVT, mirroring the immunothrombotic processes seen in sepsis and, more recently, COVID-19 [62]. The PEARL-AIS study thus situates PE risk within a global context, reinforcing the view that AIS is not merely a cerebral event but a systemic condition predisposing to life-threatening thrombotic complications. A schematic summarising the *brain–lung thromboinflammatory axis* is depicted in Figure 5.

The fact that PE carries a disproportionately high mortality burden in AIS populations is not surprising but remains clinically sobering. While population-based registries place PE case fatality between 1% and 9%, our synthesis indicates a substantially higher burden in stroke cohorts, with odds of death nearly five times greater in patients who develop PE than in those who do not [57,58]. Retrospective studies tended to report higher mortality than prospective cohorts, reflecting likely differences in detection and case mix, but the overall signal was consistent: PE in the post-stroke setting is a marker of poor prognosis. Diagnostic analyses further suggest that although PE lacks sensitivity as a predictor of mortality, its presence is highly specific for an adverse outcome, underlining its clinical gravity. Several mechanisms probably converge to explain this excess risk: impaired neurological status delays recognition of respiratory compromise [2], anticoagulation is often withheld for fear of haemorrhagic transformation [19], and the physiological reserve of patients recovering from major brain injury is diminished. Taken together, these factors suggest that PE after AIS is not a coincidental complication but a sentinel event signalling systemic decompensation and the need for urgent recognition and intervention [6,23,63].

An important insight from this analysis is the influence of study design on observed prevalence and outcomes. Retrospective datasets, often reliant on administrative coding, tended to capture more severe or fatal cases, exaggerating mortality estimates. This echoes long-standing concerns in stroke research about the limitations of coding-based epidemiology, which risks both underestimating prevalence and overestimating severity. Future studies must address this gap with protocolised surveillance, ideally integrating routine imaging in high-risk patients, to establish more reliable estimates of both prevalence and outcomes.

Regional variation in prevalence estimates may reflect differences in surveillance intensity, diagnostic resources, and reporting practices. Taken together, these patterns suggest that underdiagnosis, rather than true biological divergence, likely accounts for much of the observed geographic variation. Geographic heterogeneity in both prevalence and mortality, therefore, deserves cautious interpretation. Differences in diagnostic thresholds, access to CT pulmonary angiography, adherence to thromboprophylaxis protocols, and stroke-unit infrastructure are likely major contributors [64]. The relatively low prevalence in Asian cohorts [37,38,42] may reflect underutilisation of imaging or differences in baseline population risk, whereas the higher mortality seen in European cohorts [45,52] could indicate systematic detection of more clinically apparent or severe cases. Large North American datasets [33,36,41,46,47,49,54] may under-record fatal PE due to coding limitations, creating an artefact of lower mortality. These discrepancies highlight the urgent need for international consensus on PE definitions and surveillance practices in stroke care, ensuring comparability across health systems [65,66].

Classical predictors of venous thromboembolism performed poorly in the post-stroke setting. Male sex showed no significant association with PE, hypertension yielded inconclusive results with wide imprecision, and diabetes paradoxically appeared protective, with a consistent reduction in odds across studies. By contrast, the comorbidity profile of AIS patients who developed PE was marked by high rates of atrial fibrillation (29%), hypertension (54%), diabetes (23%), cancer (19%), and smoking (23%) (Table 2; Supplemental Information Figure S1). Cancer and smoking, both potent prothrombotic conditions [67,68], align with the concept of a *brain–lung thromboinflammatory axis* [6], whereby systemic inflammation and endothelial dysfunction overshadow classical risk factors in driving PE after AIS [6]. The counterintuitive, apparent protective signal for diabetes may reflect closer clinical surveillance, earlier mobilisation, or pharmacological effects of antidiabetic therapies such as metformin, which has been linked to anti-inflammatory and endothelial-stabilising properties. These findings challenge the primacy of traditional VTE predictors and support a model in which stroke-induced systemic alterations, not background vascular risk alone, govern the propensity for pulmonary thrombosis [6].

Pharmacological prophylaxis was consistently associated with reduced risk of PE after AIS (OR 0.64), with no heterogeneity across studies. This finding affirms what clinical practice has long assumed but rarely quantified—that pharmacological prophylaxis works [69]. The benefit was observed across different agents (danaparoid, heparin, enoxaparin), suggesting a class effect, and strengthens guideline recommendations to initiate prophylaxis in immobilised stroke patients where bleeding risk permits [29]. At the same time, important evidence gaps remain. The comparative effectiveness and safety of newer agents such as direct oral anticoagulants (DOACs) are untested in this setting, and the role of mechanical strategies such as intermittent pneumatic compression, proven for DVT but less well studied for PE, remains uncertain [29]. Addressing these gaps should be a research priority, given the disproportionate lethality of PE in stroke populations and the pressing need for preventive strategies tailored to this high-risk group.

While pooled prevalence and outcome estimates demonstrate consistent directionality, the certainty of evidence was generally low to moderate owing to considerable heterogeneity (I^2^ > 90%) and reliance on retrospective datasets. Nevertheless, the consistent protective signal for pharmacological prophylaxis (moderate-certainty evidence, I^2^ = 0%) reinforces the clinical importance of preventive strategies. The certainty of evidence, assessed using the GRADE framework (Table 5), varied across outcomes. Evidence was rated moderate for the association of PE with increased mortality, the apparent protective signal of diabetes, and the benefit of pharmacological prophylaxis, reflecting consistent effects despite limited study numbers. By contrast, certainty was low to very low for most prevalence estimates and comorbidity associations, largely due to high heterogeneity, small-study effects, and potential publication bias. This reinforces that while the PEARL-AIS study provides the most comprehensive synthesis to date, confidence in the exact figures remains limited. Clinically, however, the direction of effect is unambiguous: AIS patients are at higher risk of PE, and when PE occurs, it is often fatal. Clinicians should maintain high suspicion for PE in AIS with unexplained hypoxia and apply pharmacological prophylaxis when bleeding risk permits. The case for routine vigilance, systematic surveillance, and prophylaxis therefore remains strong, even as more definitive mechanistic and prospective studies are awaited.

## 5. Limitations

This study represents the largest and most comprehensive synthesis of pulmonary embolism after AIS to date, encompassing more than 23 million patients across diverse regions and study designs. By combining population-level administrative datasets with prospectively collected cohorts, it offers both breadth and depth, yielding robust estimates of prevalence, mortality, risk factors, and the impact of prophylaxis. Sensitivity analyses, subgroup stratification, and formal evidence grading (GRADE) further strengthen confidence in the direction of effects, even where certainty remains limited. Nonetheless, several caveats must be acknowledged. Considerable heterogeneity (I^2^ often exceeding 90%, consistent with Cochrane classification) across most analyses reflects variability in study populations, diagnostic thresholds, and reporting standards, while small numbers in many subgroups restricted robustness. Evidence for key predictors such as hypertension, diabetes, and sex was limited and imprecise, and potential publication bias could not be formally assessed. Notably, available registry data suggest that the prevalence of pulmonary embolism is lower after acute haemorrhagic stroke (approximately 0.2–0.3%) [70], although a direct comparative meta-analysis was beyond the scope of this study. Reliance on administrative datasets enhanced statistical power but introduced risks of misclassification, particularly given inconsistent use of imaging such as CT pulmonary angiography. Findings should be interpreted with caution given heterogeneity in study design and reporting quality. In particular, stroke severity measures [71] (e.g., NIHSS or Glasgow Coma Scale) and the timing or intensity of anticoagulation were often unreported, both of which could confound PE detection and outcome attribution. Incomplete reporting of reperfusion therapy and prophylaxis further constrained interpretation. To sum up, these issues underscore the need for large, prospective, harmonised studies to more precisely define the burden and determinants of PE after AIS. Methodological quality was assessed using a modified Jadad scale adapted for observational designs. The ROBINS-I tool was not applied, as most studies were retrospective or cross-sectional and did not involve interventions, randomisation, or exposure assignment. Several ROBINS-I domains, particularly those addressing intervention deviations and confounding control, were therefore inapplicable. Future extensions of the PEARL-AIS study will consider incorporating structured, domain-based bias assessment tools such as ROBINS-I or QUADAS-2 when sufficient prospective or quasi-experimental data become available.

## 6. Conclusions

Pulmonary embolism, though uncommon after AIS, carries a disproportionate mortality burden and is inadequately predicted by traditional vascular risk factors. Our synthesis indicates that atrial fibrillation, cancer, and smoking are more reliable markers, reflecting a stroke-specific *brain–lung thromboinflammatory axis* [6,72] rather than classical venous pathways [73]. Clinically, PE markedly worsens outcomes in AIS, underscoring the importance of structured surveillance in patients with unexplained hypoxia and systematic prophylaxis in immobilised or high-risk subgroups. Pharmacological prophylaxis demonstrated a consistent protective effect (moderate-certainty evidence) and should be considered standard of care where bleeding risk permits. Future prospective studies should aim to refine stroke-specific risk stratification, validate mechanistic models of thromboinflammation, and optimise preventive strategies within this evolving neurovascular–pulmonary continuum.

## Figures and Tables

**Table 2:** Pooled Prevalence, Mortality, and Risk Factors of Pulmonary Embolism After Acute Ischaemic Stroke: Summary Effects, Heterogeneity, and Significance.

Subgroup/Risk Factor	Studies (Patients) N (*n*)	Crude Prevalence (%)	Pooled Prevalence (95% CI)	I^2^ (%)	z-Score	*p*-Value
Overall prevalence of PE	17 (23,637,708)	0.48	0.40 (0.33–0.49)	99.5	16.34	<0.001
Retrospective	8 (23,606,735)	0.48	0.45 (0.36–0.54)	99.8	17.79	<0.001
Prospective	8 (30,935)	0.53	1.43 (0.59–2.58)	96.3	4.84	<0.001
Prospective + retrospective	1 (38)	21.00	–	–	–	–
Asia	3 (846,532)	0.21	0.16 (0.08–0.26)	–	5.59	<0.001
Europe	2 (2,905,657)	0.36	0.34 (0.33–0.35)	–	172.58	<0.001
Africa	1 (158)	5.69	–	–	–	–
North America	7 (19,877,825)	0.51	0.43 (0.41–0.46)	90.0	57.92	<0.001
Multiple countries	4 (7,536)	1.49	2.56 (0.76–5.21)	93.7	3.79	<0.001
Mortality in AIS patients with PE	8 (2,020,788)	0.80	12.9 (1.6–31.7)	100.0	2.77	0.006
Retrospective	6 (2,019,881)	0.80	17.16 (2.08–41.5)	99.7	2.74	0.006
Prospective	2 (907)	2.76	2.52 (1.57–3.68)	–	8.11	<0.001
Asia	3 (2,498)	3.00	2.94 (0.54–6.61)	80.1	3.08	0.002
Europe	2 (17,072)	28.59	28.59 (27.91–29.27)	0.0	145.99	<0.001
North America	2 (2,001,052)	0.56	0.31 (0.30–0.32)	0.0	105.16	<0.001
Multiple countries	1 (166)	6.02	–	–	–	–
Prevalence of risk factors in AIS patients with PE						
Atrial fibrillation	4 (17,175)	33.62	29.0 (25–35)	68.4	18.36	<0.001
Diabetes mellitus	5 (17,231)	26.03	23.0 (20–26)	55.7	23.87	<0.001
Hypertension	5 (17,231)	56.91	54.0 (49–60)	70.3	28.12	<0.001
Smoking	3 (159)	21.38	23.0 (12–37)	61.9	5.59	<0.001
Cancer	4 (17,170)	13.87	19.0 (13–25)	66.2	10.51	<0.001
Coronary artery disease	2 (10,457)	15.65	15.0 (15–16)	0.0	75.56	<0.001
Hyperlipidaemia	2 (145)	20.69	20.0 (14–27)	0.0	9.96	<0.001

This table presents pooled prevalence of pulmonary embolism (PE) after acute ischaemic stroke (AIS), stratified by study design and geographic region; pooled mortality in AIS patients with PE; and pooled prevalence of vascular and clinical risk factors in AIS patients with PE. Estimates were derived using random-effects models. Abbreviations: CI = confidence interval; I^2^ = heterogeneity statistic.

**Table 3:** Discrete predictive markers of pulmonary embolism evaluated in the PEARL-AIS meta-analysis.

Author (Year)	HTN (PE/No PE)	Cancer (PE/No PE)	AF (PE/No PE)	DM (PE/No PE)	HL (PE/No PE)	CAD (PE/No PE)	Smoking (PE/No PE)
Abdelsalam et al. (2020) [32]	–	8/1	–	–	–	–	–
Chen et al. (2012) [39]	8/6	–	6/8	1/14	–	–	5/9
Keller et al. (2024) [44]	3627/3077	1113/5591	2067/4637	1710/4994	–	–	–
Keller et al. (2020) [45]	6099/4269	1234/9134	3691/6677	2753/7615	–	1616/8752	–
Pongmoragot et al. (2013) [47]	61/28	26/63	11/78	19/70	23/66	21/67	13/76
Tanislav et al. (2011) [53]	12/44	–	–	2/54	7/49	–	16/40

Abbreviations: AF = atrial fibrillation; CAD = coronary artery disease; DM = diabetes mellitus; HL = hyperlipidaemia; HTN = hypertension; PE = pulmonary embolism.

**Table 4:** Predictive markers and outcomes associated with pulmonary embolism after acute ischaemic stroke: summary effects and heterogeneity.

Outcome	N (Studies)	*n* (Patients)	Effect Measure	Summary Effect (OR, 95%)	Test of Overall Effect (z, *p*)	Cochran’s Q	H	I^2^ * % (95% CI) ^¶^	Q *p*-Value	τ^2^ ^Φ^
Male sex	5	2,926,122	OR	0.75 (0.52–1.09)	z = –1.52, *p* = 0.129	9.82	1.57	59.3 (0.0–87.5)	0.044	0.083
Pharmacological prophylaxis	3	21,090	OR	0.64 (0.46–0.90)	z = 2.58, *p* = 0.010	0.29	0.378	0.0 (0.0–37.4)	0.867	0.000
Hypertension	3	2,933,559	OR	1.12 (0.39–3.25)	z = 0.209, *p* = 0.835	43.58	4.67	95.4 (0.0–98.9)	<0.001	0.819
Diabetes mellitus	3	2,928,800	OR	0.88 (0.84–0.92)	z = –5.97, *p* < 0.001	1.05	0.72	0.0 (0.0–58.2)	0.593	0.000
Mortality	5	3,774,118	OR	4.96 (2.98–8.24)	z = 6.18, *p* < 0.001	48.28	3.47	91.7 (0.0–97.8)	<0.001	0.270

* Values of I^2^≤ are percentages; ^¶^ eterogeneity values were calculated from data with 95% CIs based on gamma (random effects) distribution for Q; **^Φ^** Heterogeneity variance estimates (**τ**^2^≤) were derived from the DerSimonian and Laird method. Abbreviations: CI = confidence interval; H = relative excess in Cochran’s Q over degrees of freedom; I^2^ = proportion of variation due to heterogeneity; N = number of studies; n = number of patients; OR = odds ratio; REDL = DerSimonian and Laird random-effects method; Q = Cochran’s heterogeneity statistic; τ^2^ = between-study variance.

**Table 5:** GRADE Evidence Profile for Prevalence, Risk, Outcomes, and Prophylaxis of Pulmonary Embolism After Acute Ischaemic Stroke (PEARL-AIS Study).

Outcome	No. of Studies (Patients)	Effect Estimate (95% CI)	Risk of Bias	Inconsistency	Indirectness	Imprecision	Publication Bias	Certainty of Evidence (GRADE)	Reasons for Rating
Prevalence of PE after AIS	17 (23,637,708)	0.40% (95% CI 0.33–0.49)	Serious (retrospective datasets; coding bias)	Very serious (considerable heterogeneity, I^2^ > 90%)	Not serious	Serious (wide 95% CI)	Possible (funnel asymmetry)	⬤⬤◯◯ Low	Considerable heterogeneity (Cochrane classification); reliance on retrospective datasets; imprecision
Mortality in AIS patients with PE	8 (2,020,788)	12.9% (95% CI 1.6–31.7)	Serious (mixed designs; coding reliance)	Serious (considerable heterogeneity, I^2^ > 95%)	Not serious	Serious (wide 95% CI)	Undetected (few studies)	⬤⬤⬤◯ Moderate	Consistent direction but heterogeneity; limited prospective data
Mortality risk (OR)	5 (3,774,118)	OR 4.96 (95% CI 2.98–8.24)	Serious	Serious (considerable heterogeneity, I^2^ > 90%)	Not serious	Not serious (consistent large effect)	Undetected	⬤⬤⬤◯ Moderate	Large, consistent effect; down-graded for heterogeneity
Atrial fibrillation prevalence in AIS + PE	4 (17,175)	29% (95% CI 25–35)	Moderate	Serious (high heterogeneity)	Not serious	Serious (imprecise estimates)	Undetected	⬤⬤◯◯ Low	Few studies; heterogeneity across datasets
Cancer prevalence in AIS + PE	4 (17,170)	19% (95% CI 13–25)	Moderate	Serious (reporting variation)	Not serious	Serious (wide 95% CI)	Undetected	⬤⬤◯◯ Low	Limited data; possible under-reporting
Smoking prevalence in AIS + PE	3 (159)	23% (95% CI 12–37)	Moderate	Serious (high heterogeneity)	Not serious	Very serious (small sample; wide 95% CI)	Undetected	⬤◯◯◯ Very low	Small cohorts; wide confidence interval
Diabetes as predictor of PE	3 (2,928,800)	OR 0.88 (95% CI 0.84–0.92)	Low	Not serious (I^2^ = 0%)	Not serious	Not serious (narrow 95% CI)	Unlikely	⬤⬤⬤◯ Moderate	Consistent effect, no heterogeneity; inverse association
Hypertension as predictor of PE	3 (2,933,559)	OR 1.12 (95% CI 0.39–3.25)	Moderate	Very serious (considerable heterogeneity, I^2^ > 95%)	Not serious	Serious (wide 95% CI)	Possible	⬤◯◯◯ Very low	Extreme heterogeneity; imprecision of effect
Pharmacological prophylaxis	3 (21,090)	OR 0.64 (95% CI 0.46–0.90)	Low	Not serious (I^2^ = 0%)	Not serious	Serious (few studies)	Undetected	⬤⬤⬤◯ Moderate	Consistent protective effect; no heterogeneity; limited sample size

The GRADE table summarises the certainty of evidence for prevalence, outcomes, risk factors, and prophylaxis of pulmonary embolism (PE) after acute ischaemic stroke (AIS). Certainty was assessed across five domains (risk of bias, inconsistency, indirectness, imprecision, publication bias). Evidence was graded as high (⬤⬤⬤⬤), moderate (⬤⬤⬤◯), low (⬤⬤◯◯), or very low (⬤◯◯◯). Predictors without significant associations (e.g., male sex) were not included. Abbreviations: AIS = acute ischaemic stroke; CI = confidence interval; OR = odds ratio; PE = pulmonary embolism; GRADE = Grading of Recommendations, Assessment, Development and Evaluation.

## Data Availability

The original contributions/data and analyses presented in this study are included in the article and online Supplementary Information. Further inquiries can be directed to the corresponding author.

## Supplementary Materials

The following supporting information can be downloaded at: https://www.mdpi.com/article/10.3390/neurolint17100168/s1, Figure S1: Forest Plots of Discrete Predictive Indicators of Pulmonary Embolism; Figure S2: Graphs of Egger’s Regression Test for Meta-analysis on the Association between Predictive Indicators or Clinical Outcomes and Pulmonary Embolism; Figure S3: Sensitivity Analysis on Association between Predictive Indicators or Clinical Outcomes and Pulmonary Embolism; Table S1: PRISMA 2020 checklist for the PEARL-AIS systematic review and meta-analysis; Table S2: MOOSE Checklist for the PEARL-AIS systematic review and meta-analysis; Table S3: Methodological Quality Assessment using Modified Jadad Analysis; Table S4: Funding Bias Scores for Studies; Table S5: Outputs from Egger’s Test for Publication Bias; Table S6: Diagnostic Modality and Follow-up Window of PE in studies included in the PEARL-AIS analysis; Table S7: Summary of Pharmacological Intervention Characteristics Across Included Studies.
