# Supplementary material for: Pulmonary Embolism After Acute Ischaemic Stroke (PEARL-AIS): Global Prevalence, Risk Factors, Outcomes, and Evidence Grading from a Meta-Analysis
Source: Neurol Int. 2025 Oct 12;17(10):168. doi: 10.3390/neurolint17100168 (PMC12567275; doi:10.3390/neurolint17100168)
Supplement: Supplementary file 1 [file neurolint-17-00168-s001.zip › neurolint-3893468-supplementary.pdf]

SUPPLEMENTAL INFORMATION

Pulmonary Embolism After Acute Ischaemic Stroke (PEARL-AIS): Global Prevalence, Risk Factors, Outcomes, and Evidence Grading from a Meta-Analysis

Supplementary Information – Table of Contents

1. **Search Strategy (Keywords/MeSH Terms)**
   - **a. PubMed Search Strategy**
   - **b. Embase Search Strategy**
   - **c. Cochrane Library Search Strategy**
   - **d. Scopus Search Strategy**
   - **e. Web of Science Search Strategy**
   - **f. Other Sources**
2. **Supplemental Figures**
   - **a. Supplemental Figure S1:** Forest Plots of Discrete Predictive Indicators of Pulmonary Embolism
   - **b. Supplemental Figure S2:** Graphs of Egger's Regression Test for Meta-analysis on the Association between Predictive Indicators or Clinical Outcomes and Pulmonary Embolism
   - **c. Supplemental Figure S3:** Sensitivity Analysis on Association between Predictive Indicators or Clinical Outcomes and Pulmonary Embolism

3. **Supplemental Tables**
   - **a. Supplemental Table S1:** PRISMA-2020 Checklist for the PEARL-AIS systematic review and meta-analysis
   - **b. Supplemental Table S2:** MOOSE Checklist for the PEARL-AIS systematic review and meta-analysis
   - **c. Supplemental Table S3:** Methodological Quality Assessment using the Modified Jadad Analysis
   - **d. Supplemental Table S4:** Funding Bias Scores for Studies
   - **e. Supplemental Table S5:** Outputs from Egger's Test for Publication Bias
   - **f. Supplemental Table S6:** Diagnostic Modality and Follow-up Window of PE in studies included in the PEARL-AIS analysis.
   - **g. Supplemental Table S7:** Summary of Pharmacological Intervention Characteristics Across Included Studies

1. Search Strategy

a. PubMed Search Strategy

Search Terms and Boolean Operators:

1. **PE Incidence in AIS Patients**
   ("pulmonary embolism" OR "PE") AND ("acute ischemic stroke" OR "AIS") AND ("incidence")
2. **Risk Factors for PE in AIS Patients**
   ("pulmonary embolism" OR "PE") AND ("acute ischemic stroke" OR "AIS") AND ("risk factors")
3. **Prophylactic Strategies for PE in AIS Patients**
   ("pulmonary embolism" OR "PE") AND ("acute ischemic stroke" OR "AIS") AND ("prophylactic prevention" OR "pharmacological agents" OR "mechanical devices")
4. **PE in AIS Patients Undergoing Reperfusion Therapy**
   ("pulmonary embolism" OR "PE") AND ("acute ischemic stroke" OR "AIS") AND ("intravenous thrombolysis" OR "IVT" OR "endovascular thrombectomy" OR "EVT")
5. **Effectiveness of Prophylactic Interventions**
   ("pulmonary embolism" OR "PE") AND ("acute ischemic stroke" OR "AIS") AND ("effectiveness" OR "prevention strategies" OR "pharmacological" OR "mechanical")

Filters applied: Clinical Trial, Meta-Analysis, Observation Study, Randomised Controlled Trial, Review, Systematic Review, English, Adult: 19+ years

Results: 249

b. Embase Search Strategy

Search Terms and Boolean Operators:

1. **PE Incidence in AIS Patients**
   ("pulmonary embolism" OR "PE") AND ("acute ischemic stroke" OR "AIS") AND ("incidence")
2. **Risk Factors for PE in AIS Patients**
   ("pulmonary embolism" OR "PE") AND ("acute ischemic stroke" OR "AIS") AND ("risk factors")
3. **Prophylactic Strategies for PE in AIS Patients**
   ("pulmonary embolism" OR "PE") AND ("stroke") AND ("prophylactic prevention" OR "pharmacological agents" OR "mechanical devices")
4. **PE in AIS Patients Undergoing Reperfusion Therapy**
   ("pulmonary embolism" OR "PE") AND ("acute ischemic stroke" OR "AIS") AND ("intravenous thrombolysis" OR "IVT" OR "endovascular thrombectomy" OR "EVT")
5. **Effectiveness of Prophylactic Interventions**
   ("pulmonary embolism" OR "PE") AND ("acute ischemic stroke" OR "AIS") AND ("effectiveness" OR "prevention strategies" OR "pharmacological" OR "mechanical")

Filters: 18-64 years, 65+ years, Human, English

Results: 322

c. **Cochrane Library Search Strategy**

Search Terms and Boolean Operators:

1. **PE Incidence in AIS Patients**
("pulmonary embolism" OR "PE") AND ("acute ischemic stroke" OR "AIS") AND ("incidence")
2. **Risk Factors for PE in AIS Patients**
("pulmonary embolism" OR "PE") AND ("acute ischemic stroke" OR "AIS") AND ("risk factors")
3. **Prophylactic Strategies for PE in AIS Patients**
("pulmonary embolism" OR "PE") AND ("stroke") AND ("prophylactic prevention" OR "pharmacological agents" OR "mechanical devices")
4. **PE in AIS Patients Undergoing Reperfusion Therapy**
("pulmonary embolism" OR "PE") AND ("acute ischemic stroke" OR "AIS") AND ("intravenous thrombolysis" OR "IVT" OR "endovascular thrombectomy" OR "EVT")
5. **Effectiveness of Prophylactic Interventions**
("pulmonary embolism" OR "PE") AND ("acute ischemic stroke" OR "AIS") AND ("effectiveness" OR "prevention strategies" OR "pharmacological" OR "mechanical")

Filters: English

Results: 63

d. **Scopus Search Strategy**

Search Terms and Boolean Operators:

1. **PE Incidence in AIS Patients**
("pulmonary embolism" OR "PE") AND ("acute ischemic stroke" OR "AIS") AND ("incidence")
2. **Risk Factors for PE in AIS Patients**
("pulmonary embolism" OR "PE") AND ("acute ischemic stroke" OR "AIS") AND ("risk factors")
3. **Prophylactic Strategies for PE in AIS Patients**
("pulmonary embolism" OR "PE") AND ("stroke") AND ("prophylactic prevention" OR "pharmacological agents" OR "mechanical devices")
4. **PE in AIS Patients Undergoing Reperfusion Therapy**
("pulmonary embolism" OR "PE") AND ("acute ischemic stroke" OR "AIS") AND ("intravenous thrombolysis" OR "IVT" OR "endovascular thrombectomy" OR "EVT")

5. **Effectiveness of Prophylactic Interventions**
   ("pulmonary embolism" OR "PE") AND ("acute ischemic stroke" OR "AIS") AND ("effectiveness" OR "prevention strategies" OR "pharmacological" OR "mechanical")

Filters: Human, English

Results: 190

e. Web of Science Search Strategy

Search Terms and Boolean Operators:

1. **PE Incidence in AIS Patients**
   ("pulmonary embolism" OR "PE") AND ("acute ischemic stroke" OR "AIS") AND ("incidence")
2. **Risk Factors for PE in AIS Patients**
   ("pulmonary embolism" OR "PE") AND ("acute ischemic stroke" OR "AIS") AND ("risk factors")
3. **Prophylactic Strategies for PE in AIS Patients**
   ("pulmonary embolism" OR "PE") AND ("acute ischemic stroke" OR "AIS") AND ("prophylactic prevention" OR "pharmacological agents" OR "mechanical devices")
4. **PE in AIS Patients Undergoing Reperfusion Therapy**
   ("pulmonary embolism" OR "PE") AND ("acute ischemic stroke" OR "AIS") AND ("intravenous thrombolysis" OR "IVT" OR "endovascular thrombectomy" OR "EVT")
5. **Effectiveness of Prophylactic Interventions**
   ("pulmonary embolism" OR "PE") AND ("acute ischemic stroke" OR "AIS") AND ("effectiveness" OR "prevention strategies" OR "pharmacological" OR "mechanical")

Filters: English

Results: 384

f. Other Sources

Additional sources were identified through hand-searching and Google Scholar.

Results: 12

2. Supplemental Figures

a. **Supplemental Figure S1:** Forest Plots of Discrete Predictive Indicators of Pulmonary Embolism

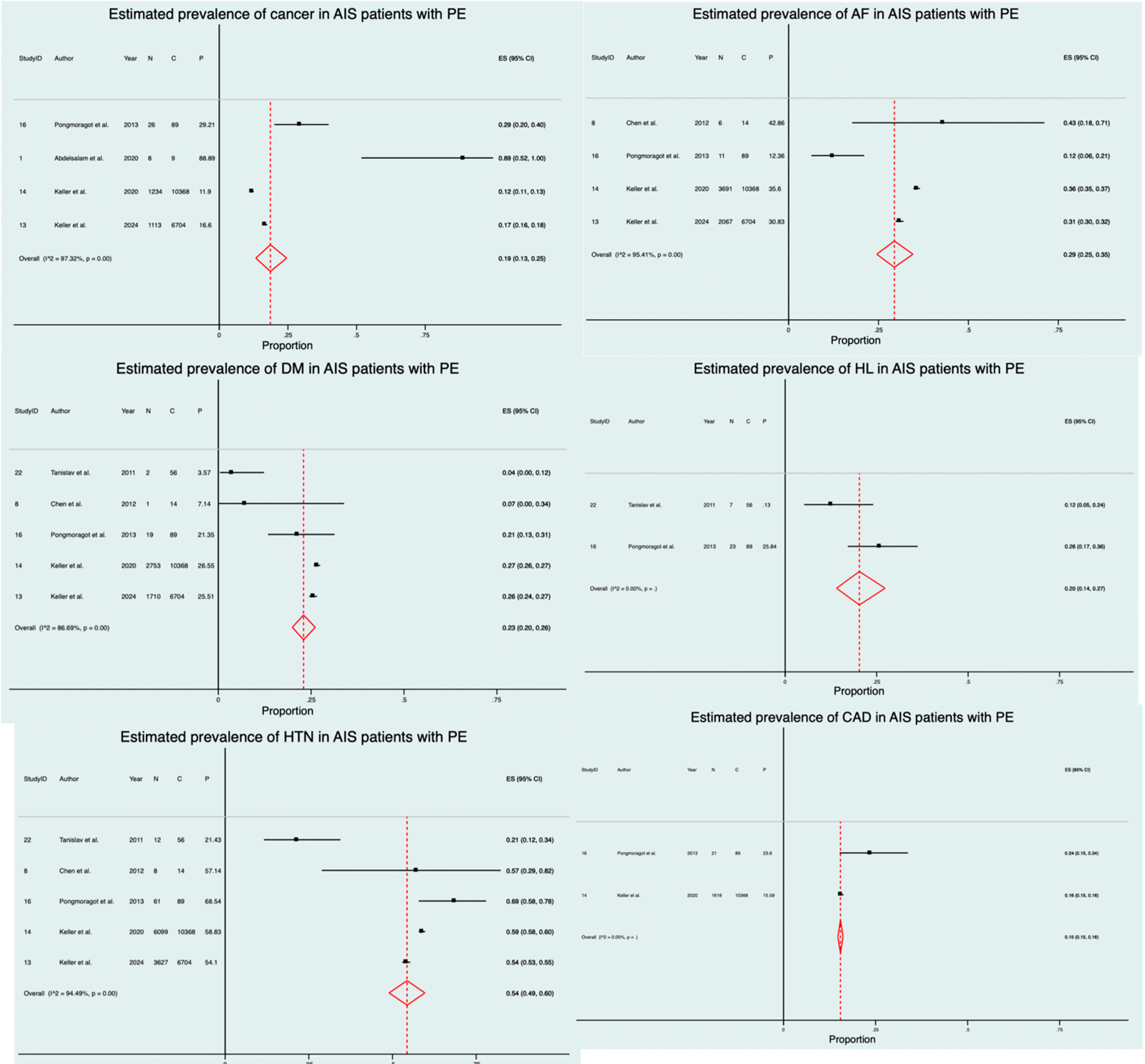

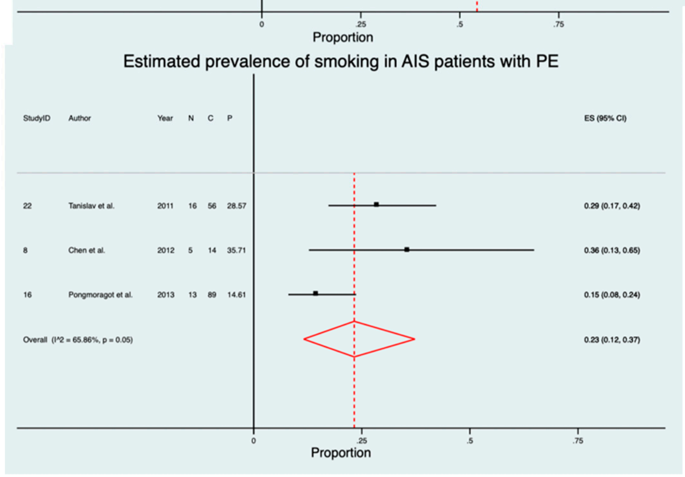

Abbreviations: AF: atrial fibrillation; AIS: acute ischaemic stroke; C: total number of patients; CAD: coronary artery disease; CI: confidence interval; DM: diabetes mellitus; ES: effect size; HL: hyperlipidaemia; HTN: hypertension; P: proportion of patients with outcome; PE: pulmonary embolism; N: number of patients with outcome

b. **Supplemental Figure S2:** Graphs of Egger's Regression Test for Meta-analysis on the Association between Predictive Indicators or Clinical Outcomes and Pulmonary Embolism

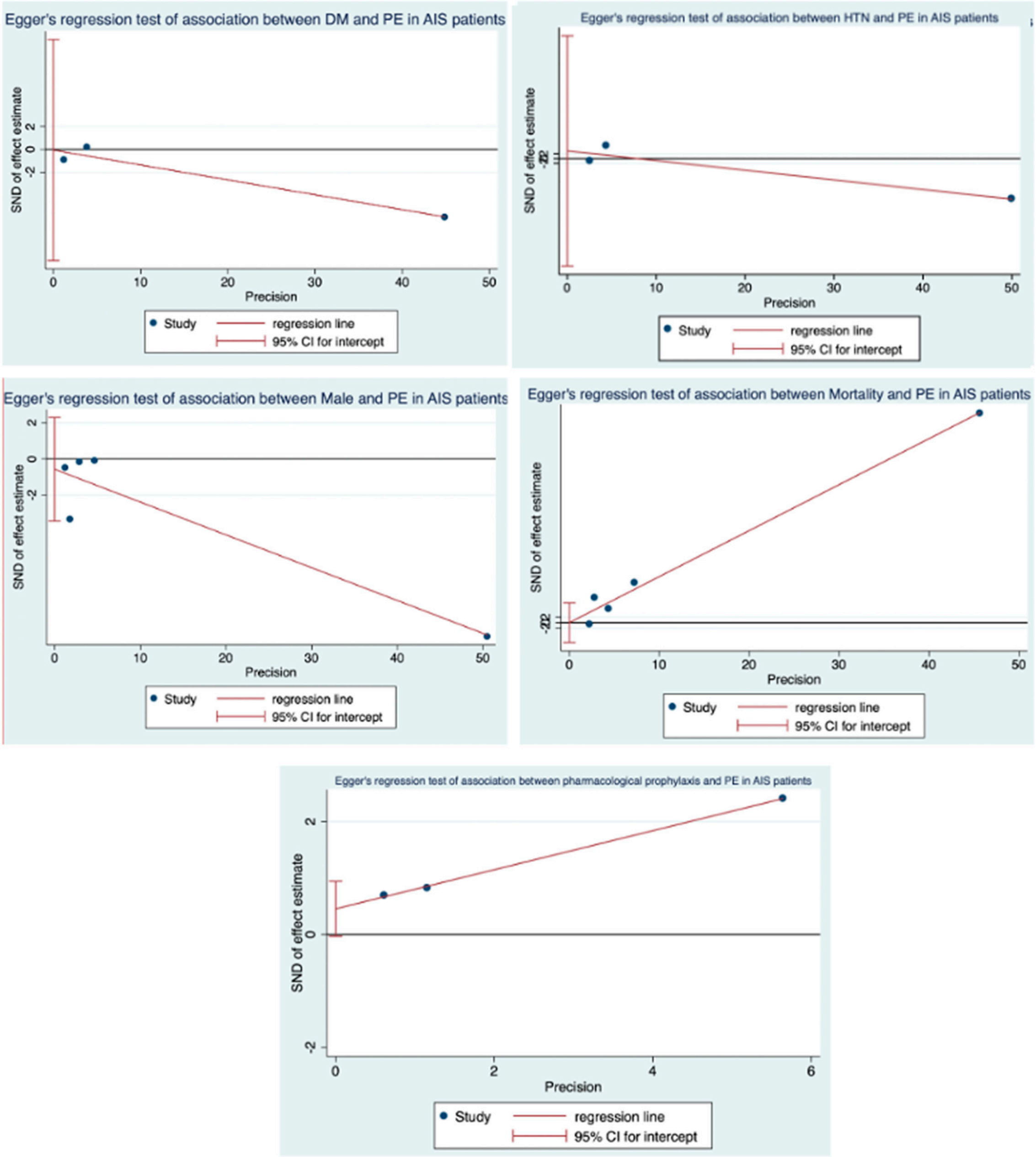

c. Supplemental Figure S3: Sensitivity Analysis on Association between Predictive Indicators or Clinical Outcomes and Pulmonary Embolism

a) Male

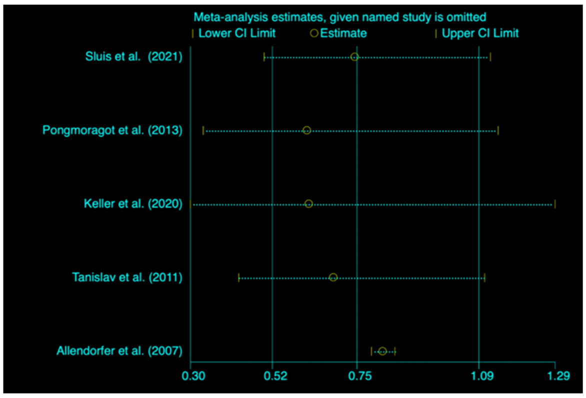

b) Hypertension

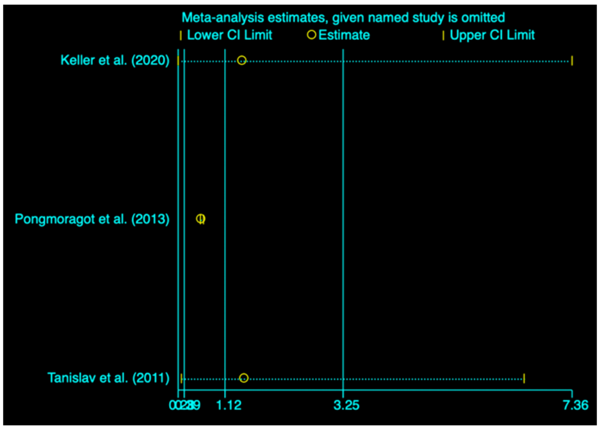

c) Diabetes Mellitus

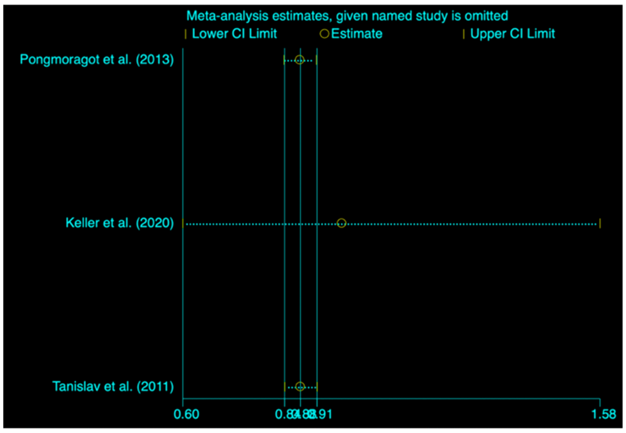

d) Mortality

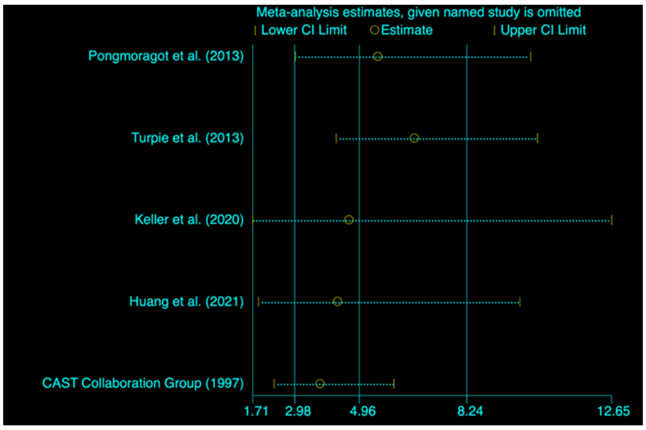

e) Pharmacological Prophylaxis

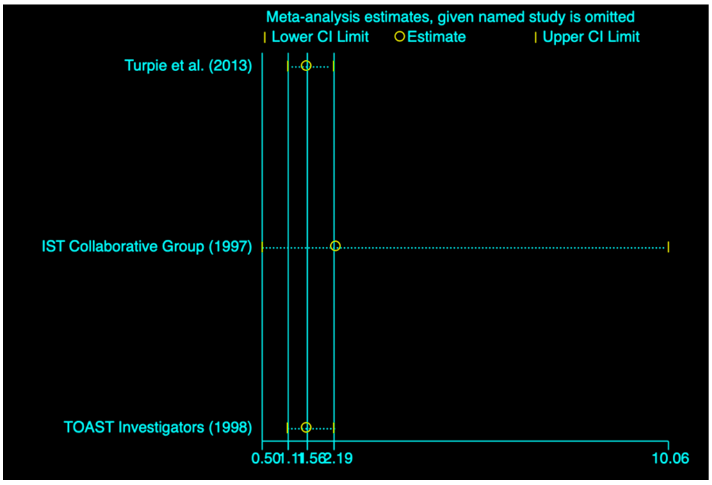

3. Supplemental Tables

a. Supplemental Table S1: *PRISMA 2020 checklist for the PEARL-AIS systematic review and meta-analysis*

Section and Topic	Item #	Checklist item	Location where item is reported
**TITLE**			
Title	1	Identify the report as a systematic review.	1
**ABSTRACT**			
Abstract	2	See the PRISMA 2020 for Abstracts checklist.	3
**INTRODUCTION**			
Rationale	3	Describe the rationale for the review in the context of existing knowledge.	4
Objectives	4	Provide an explicit statement of the objective(s) or question(s) the review addresses.	5
**METHODS**			
Eligibility criteria	5	Specify the inclusion and exclusion criteria for the review and how studies were grouped for the syntheses.	6
Information sources	6	Specify all databases, registers, websites, organisations, reference lists and other sources searched or consulted to identify studies. Specify the date when each source was last searched or consulted.	5, Supplemental Information
Search strategy	7	Present the full search strategies for all databases, registers and websites, including any filters and limits used.	Supplemental Information
Selection process	8	Specify the methods used to decide whether a study met the inclusion criteria of the review, including how many reviewers screened each record and each report retrieved, whether they worked independently, and if applicable, details of automation tools used in the process.	6
Data collection process	9	Specify the methods used to collect data from reports, including how many reviewers collected data from each report, whether they worked independently, any processes for obtaining or confirming data from study investigators, and if applicable, details of automation tools used in the process.	6
Data items	10a	List and define all outcomes for which data were sought. Specify whether all results that were compatible with each outcome domain in each study were sought (e.g. for all measures, time points, analyses), and if not, the	6, Supplemental Information

Section and Topic	Item #	Checklist item	Location where item is reported
		methods used to decide which results to collect.	
	10b	List and define all other variables for which data were sought (e.g. participant and intervention characteristics, funding sources). Describe any assumptions made about any missing or unclear information.	6, Supplemental Information
Study risk of bias assessment	11	Specify the methods used to assess risk of bias in the included studies, including details of the tool(s) used, how many reviewers assessed each study and whether they worked independently, and if applicable, details of automation tools used in the process.	6
Effect measures	12	Specify for each outcome the effect measure(s) (e.g. risk ratio, mean difference) used in the synthesis or presentation of results.	6
Synthesis methods	13a	Describe the processes used to decide which studies were eligible for each synthesis (e.g. tabulating the study intervention characteristics and comparing against the planned groups for each synthesis (item #5)).	N/A
	13b	Describe any methods required to prepare the data for presentation or synthesis, such as handling of missing summary statistics, or data conversions.	7
	13c	Describe any methods used to tabulate or visually display results of individual studies and syntheses.	7
	13d	Describe any methods used to synthesize results and provide a rationale for the choice(s). If meta-analysis was performed, describe the model(s), method(s) to identify the presence and extent of statistical heterogeneity, and software package(s) used.	7
	13e	Describe any methods used to explore possible causes of heterogeneity among study results (e.g. subgroup analysis, meta-regression).	7
	13f	Describe any sensitivity analyses conducted to assess robustness of the synthesized results.	7
Reporting bias assessment	14	Describe any methods used to assess risk of bias due to missing results in a synthesis (arising from reporting biases).	6
Certainty assessment	15	Describe any methods used to assess certainty (or confidence) in the body of evidence for an outcome.	6

Section and Topic	Item #	Checklist item	Location where item is reported
**RESULTS**			
Study selection	16a	Describe the results of the search and selection process, from the number of records identified in the search to the number of studies included in the review, ideally using a flow diagram.	Figure 1
	16b	Cite studies that might appear to meet the inclusion criteria, but which were excluded, and explain why they were excluded.	Figure 1
Study characteristics	17	Cite each included study and present its characteristics.	Table 1-2
Risk of bias in studies	18	Present assessments of risk of bias for each included study.	Supplemental Information
Results of individual studies	19	For all outcomes, present, for each study: (a) summary statistics for each group (where appropriate) and (b) an effect estimate and its precision (e.g. confidence/credible interval), ideally using structured tables or plots.	Table 3-6
Results of syntheses	20a	For each synthesis, briefly summarise the characteristics and risk of bias among contributing studies.	7-9, Supplemental Information
	20b	Present results of all statistical syntheses conducted. If meta-analysis was done, present for each the summary estimate and its precision (e.g. confidence/credible interval) and measures of statistical heterogeneity. If comparing groups, describe the direction of the effect.	7-9, Table 3-6, Supplemental Information
	20c	Present results of all investigations of possible causes of heterogeneity among study results.	7-9, Table 3-6, Supplemental Information
	20d	Present results of all sensitivity analyses conducted to assess the robustness of the synthesized results.	N/A
Reporting biases	21	Present assessments of risk of bias due to missing results (arising from reporting biases) for each synthesis assessed.	7-9, Supplemental Information
Certainty of evidence	22	Present assessments of certainty (or confidence) in the body of evidence for each outcome assessed.	7-9, Supplemental Information

Section and Topic	Item #	Checklist item	Location where item is reported
**DISCUSSION**			
Discussion	23a	Provide a general interpretation of the results in the context of other evidence.	10-12
	23b	Discuss any limitations of the evidence included in the review.	10-13
	23c	Discuss any limitations of the review processes used.	13-14
	23d	Discuss implications of the results for practice, policy, and future research.	10-14
**OTHER INFORMATION**			
NA	24a	Provide registration information for the review, including register name and registration number, or state that the review was not registered.	5
	24b	Indicate where the review protocol can be accessed, or state that a protocol was not prepared.	N/A
	24c	Describe and explain any amendments to information provided at registration or in the protocol.	N/A
Support	25	Describe sources of financial or non-financial support for the review, and the role of the funders or sponsors in the review.	N/A
Competing interests	26	Declare any competing interests of review authors.	14
Availability of data, code and other materials	27	Report which of the following are publicly available and where they can be found: template data collection forms; data extracted from included studies; data used for all analyses; analytic code; any other materials used in the review.	Supplemental Information

From: [1] Page MJ, McKenzie JE, Bossuyt PM, Boutron I, Hoffmann TC, Mulrow CD, et al. The PRISMA 2020 statement: an updated guideline for reporting systematic reviews. BMJ 2021;372:n71. doi: 10.1136/bmj

b. **Supplemental Table S2**: *MOOSE Checklist for the PEARL-AIS systematic review and meta-analysis*

Item No	Recommendation	Reported on Page No
Reporting of background should include		
1	Problem definition	4
2	Hypothesis statement	5
3	Description of study outcome(s)	5
4	Type of exposure or intervention used	5
5	Type of study designs used	5
6	Study population	6
Reporting of search strategy should include		
7	Qualifications of searchers (eg, librarians and investigators)	1
8	Search strategy, including time period included in the synthesis and key words	5, Supplemental Information
9	Effort to include all available studies, including contact with authors	5
10	Databases and registries searched	5, Supplemental Information
11	Search software used, name and version, including special features used (eg, explosion)	5, Supplemental Information
12	Use of hand searching (eg, reference lists of obtained articles)	5, Supplemental Information
13	List of citations located and those excluded, including justification	Figure 1
14	Method of addressing articles published in languages other than English	N/A
15	Method of handling abstracts and unpublished studies	N/A
16	Description of any contact with authors	N/A
Reporting of methods should include		
17	Description of relevance or appropriateness of studies assembled for assessing the hypothesis to be tested	5
18	Rationale for the selection and coding of data (eg, sound clinical principles or convenience)	5
19	Documentation of how data were classified and coded (eg, multiple raters, blinding and interrater reliability)	5
20	Assessment of confounding (eg, comparability of cases and controls in studies where appropriate)	N/A
21	Assessment of study quality, including blinding of quality assessors, stratification or regression on possible predictors of study results	5
22	Assessment of heterogeneity	Supplemental Information
23	Description of statistical methods (eg, complete description of fixed or random effects models, justification of whether the chosen models account for predictors of study results, dose-response models, or cumulative meta-analysis) in sufficient detail to be replicated	7

24	Provision of appropriate tables and graphics	Tables 1-7, Figures 1-4
Reporting of results should include		
25	Graphic summarizing individual study estimates and overall estimate	Figures 2-4
26	Table giving descriptive information for each study included	Table 1
27	Results of sensitivity testing (eg, subgroup analysis)	Tables 3-4
28	Indication of statistical uncertainty of findings	N/A
**Item No**	**Recommendation**	**Reported on Page No**
Reporting of discussion should include		
29	Quantitative assessment of bias (eg, publication bias)	Supplemental Information
30	Justification for exclusion (eg, exclusion of non-English language citations)	5, Figure 1
31	Assessment of quality of included studies	6, Supplemental Information
Reporting of conclusions should include		
32	Consideration of alternative explanations for observed results	10-14
33	Generalization of the conclusions (ie, appropriate for the data presented and within the domain of the literature review)	10-14
34	Guidelines for future research	13-14
35	Disclosure of funding source	15

From: Stroup DF, Berlin JA, Morton SC, et al, for the Meta-analysis Of Observational Studies in Epidemiology (MOOSE) Group. Meta-analysis of Observational Studies in Epidemiology. A Proposal for Reporting. JAMA. 2000;283(15):2008-2012. doi: 10.1001/jama.283.15.2008.

c. **Supplemental Table S3**: Methodological Quality Assessment using *Modified Jadad Analysis*

StudyID	Authors	Criteria 1	Criteria 2	Criteria 3	Criteria 4	Criteria 5	Criteria 6	Criteria 7	Criteria 8	Total
1	Abdelsalam et al.	0	0	0	0	0	1	1	1	3
2	Ahmed et al	0	0	0	0	0	1	1	1	3
3	Ali et al.	0	0	0	0	0	1	1	1	3
4	Allendorfer et al.	0	0	0	0	0	0	1	0	1
5	Amin et al.	0	0	0	0	0	1	1	0	2
6	CAST Collaboration Group	1	1	0	0	1	1	1	1	6
7	Che et al.	0	0	0	0	0	1	1	1	2
8	Chen et al.	0	0	0	0	0	1	1	1	3
9	Dennis et al.	1	1	0.5	1	1	1	1	1	7.5
10	Eswaradass et al.	0	0	0	0	0	1	1	0	2
11	Huang et al.	0	0	0	0	0	1	1	1	3
12	IST Collaborative Group	1	1	0	0	1	1	1	1	6
13	Keller et al.	0	0	0	0	0	1	1	1	3
14	Keller et al.	0	0	0	0	0	1	1	1	3
15	Kelly et al.	0	0	0	0	0	1	1	1	3
16	Pongmoragot et al.	0	0	0	0	0	1	1	1	3
17	Sherman et al.	1	1	0	0	1	1	1	1	6
18	Skaf et al.	0	0	0	0	0	0	1	1	2
19	Skaf et al.	0	0	0	0	0	0	1	1	3
20	Sluis et al.	0	0	0	0	0	1	1	1	3
21	Sprigg et al.	1	1	1	1	0	1	1	1	7

22	Tanislav et al.	0	0	0	0	0	1	1	1	3
23	TOAST Investigators	1	1	1	1	1	1	1	1	8
24	Turpie et al.	1	1	1	1	1	1	1	1	8

Criteria 1: Was the study randomised? (0 = not described or no, 1 = yes)
Criteria 2: Was the method of randomisation appropriate (0 = not described or no, 1 = yes)
Criteria 3: Was the study described as being blinded? (0 = not described or no, 0.5 = single blinded 1 = double-blinded)
Criteria 4: Was the method of blinding appropriate (0 = not described or no, 1 = yes)
Criteria 5: Was there a description of withdrawals and dropouts? (0 = not described or no, 1 = yes)
Criteria 6: Was there a clear description of the inclusion/exclusion criteria? (0 = not described or no, 1 = yes)
Criteria 7: Was the method used to assess adverse events described? (0 = not described or no, 1 = yes)
Criteria 8: Was the method of statistical analysis described? (0 = not described or no, 1 = yes)

d. **Supplemental Table S4:** *Funding Bias Scores for Studies*

StudyID	Authors	Publication Bias	Funding
1	Abdelsalam et al.	0	No conflicts of interest
2	Ahmed et al.	0	No conflicts of interest
3	Ali et al.	0	No conflicts of interest
4	Allendorfer et al.	0	No conflicts of interest
5	Amin et al.	2	Supported by Sanofi U,S, Inc and Quintiles Consulting
6	CAST Collaboration Group	1	Supported by the Medical Research Council, Shandong Xinhua Pharmaceuticals. Oxford Department of Biochemistry.
7	Che et al.	1	Supported by the National Natural Science Foundation of China and the Beijing Natural Science Foundation.
8	Chen et al.	0	No conflicts of interest
9	Dennis et al.	0	Supported by Chief Scientist Office of the Scottish Government, Chest Heart and Stroke Scotland, Medical Research Council of the UK, Covidien, NHS Research Scotland, Department of Health UK.
10	Eswaradass et al.	0	No conflict of interest
11	Huang et al.	1	Supported by the Ministry of Science and Technology of the People's Republic of China, National Natural Science Foundation of China, Beijing Talents Project, Youth Programme, CAMS Innovation Fund for Medical Sciences, Beijing Municipal Committee of Science and Technology, Beijing Natural Science Foundation.
12	IST Collaborative Group	1	Supported in UK by the UK Medical Research Council, UK Stroke Association, and the European Union BIOMED-1 programme, Eli Lilly, Sterling Winthrop, Sanofi, and Bayer UK. Supported in Australia by the National Heart Foundation, in Canada by Nova Scotia Heart and Stroke Foundation Supported in Czech Republic IST by IGA Ministry of Health. Supported in India IST by McMaster INCLEN program and the All India Institute of Medical Sciences. Supported in New Zealand by the Julius Brendel Trust and the Lottery Grants Board, and in Norway by the Norwegian Council on Cardiovascular Disease and Nycomed Some authors received honoraria to lecture at pharmaceutical industry conferences.
13	Keller et al.	1	Supported by the German Federal Ministry of Education and Research. An author received lecture honoraria from MSD. An author received consultancy and lecture honoraria from Actelion, Bayer, Daiichi-Sankyo, MSD, Pfizer – Bristol-Myers Squibb and research funding from BRAHMS.

14	Keller et al.	2	An author received lecture and consultant fees from John & Johnson, MSD, Boston Scientific and Inari Medical
15	Kelly et al..	0	No conflicts of interest
16	Pongmoragot et al.	0	Supported by the Institute of Clinical Evaluative Sciences of the Ontario Ministry of Health and Long-term Care.
17	Sherman et al.	2	An author has received honoraria from Sanofi-Aventis for speaker bureau and consultancy. An author has received honoraria for membership of speaker bureaus for Boehringer-Ingelheim and Sanofi-Aventis, and from Organon for consultancy. An author received honoraria from Sanofi-Aventis, Pfizer, BMS, and Leo for consultancy.
18	Skaf et al.	0	No conflicts of interest
19	Skaf et al.	0	No conflicts of interest
20	Sluis et al.	1	An author received research support from the European Union's Horizon 2020 Research and Innovation Programmae, an author is supported by the University College London, an aother received a grant from the Dutch Heart Foundation and ZonMw/NWO, an author received grants from Stryker, ZonMW.
21	Sprigg et al	0	No conflict of interest
22	Tanislav et al.	0	No conflicts of interest
23	TOAST Investigators	1	Supported by US Public Health Service, National Institute of Health, National Institute of Neurological Disorders and Stroke. Supported by Organon Inc.,
24	Turpie et al.	2	Supported by Sanofi. An author received speaker's bureau fees from Bayer, BMS/Pfizer, Boehringer-Ingelheim, J&J, GSK and Sanofi, an author received honoraria from Sanofi, Leo Pharma, GSK, Bayer, Pfizer and research grants from Sanofi and Leo Pharma. An author received grant support from Sanofi and Bayer.

0 = Low potential for bias
1 = Conflicts of interest declared relating to industry funding outside of current research publication
2 = Funded by industry
3 = High potential for bias

e. **Supplemental Table S5:** Outputs from Egger's Test for Publication Bias

Outcome	Std_Eff	Coefficient [95% CI]	Standard Error	t	P > | t |	Test of H0: no small-study effects
Male	Slope	-0.18 [-0.307; -0.056]	0.04	-4.59	0.019	0.571
Male	Bias	-0.57 [-3.440; 2.298]	0.90	-0.63	0.571	
HTN	Slope	-0.41 [-2.098; 1.276]	0.13	-3.19	0.199	0.554
HTN	Bias	3.24 [-45.71; 52.20]	3.85	0.84	0.554	
DM	Slope	-0.13 [-0.505; 0.243]	0.03	-4.45	0.141	0.949
DM	Bias	-0.06 [-9.796; 9.674]	0.76	-0.08	0.949	
Mortality	Slope	1.65 [1.312; 1.996]	0.11	15.39	0.001	0.985
Mortality	Bias	-0.04 [-7.170; 7.080]	2.24	-0.02	0.985	
Pharm	Slope	0.35 [0.200; 0.491]	0.01	30.18	0.021	0.053

Abbreviations: CI: confidence interval; DM: diabetes mellitus; HTN: hypertension

f. Supplemental Table S6. Diagnostic Modality and Follow-up Window of PE in studies included in the PEARL-AIS analysis.

Author (Year)	PE diagnosis modality	Follow-up window
Abdelsalam et al. (2020)[31]	Database	90 days
Ahmed et al. (2023)[32]	ICD code	In-hospital only
Ali et al. (2009)[36]	database	30 and 90 days
Allendorfer et al. (2007)[53]	V/Q scan	3.5 years
Amin et al. (2013)[33]	ICD code	30 days
CAST Collaboration Group (1997)[46]	clinical	30 days
Che et al. (2024)[44]	ICD code	90 days
Chen et al. (2012)[50]	V/Q scan or helical CT	-
Dennis et al. (2011)[38]	V/Q scan or CTPA	30 days
Eswaradass et al. (2018)[41]	CTPA	In-hospital only
Huang et al. (2021)[42]	database	In-hospital only
IST Collaborative Group (1997)[54]	Medical records	14 days
Keller et al. (2024)[49]	ICD code	In-hospital only
Keller et al. (2020)[43]	ICD code	In-hospital only
Kelly et al. (2004)[34]	clinical	In-hospital
Pongmoragot et al. (2013)[37]	CTPA	30 days
Sherman et al. (2007)[39]	V/Q scan or helical CT	14 days
Skaf et al. (2005)[45]	ICD code	In-hospital only
Skaf et al. (2006)[51]	ICD code	In-hospital only
Sluis et al. (2021)[35]	database	In-hospital only
Sprigg et al. (2005)[40]	V/Q scan	15 days
Tanislav et al. (2011)[52]	V/Q scan	In-hospital only
TOAST Investigators (1998)[47]	clinical	90 days
Turpie et al. (2013)[48]	V/Q scan or CTPA	14 and 28 days

Abbreviations: CT = computed tomography; CTPA = computed tomography pulmonary angiography; ICD = International Classification of Diseases; V/Q = ventilation perfusion

g. Supplemental Table S7. Summary of Pharmacological Intervention Characteristics Across Included Studies

Study	Agent	Dose	Intention	Duration	PE diagnosis modality
IST[54]	Heparin	5000 or 12000 IU bd	Therapeutic for AIS, prophylactic for PE	90 days	Medical records
TOAST[47]	Danaparoid	IV infusion 0.6-0.8U/mL of anti-factor Xa	Therapeutic for AIS, prophylactic for PE	In-hospital only	Clinical
EXCLAIM[48]	Enoxaparin	40mg bd	Prophylactic	30 and 90 days	V/Q scan or CTPA

Abbreviations: AIS = acute ischaemic stroke; bd = twice daily; CTPA = computed tomography pulmonary angiography; IV = intravenous; PE = pulmonary embolism; V/Q = ventilation perfusion
